# Trapping DNA Radicals With DMPO Reduces Hypochlorous Acid-Induced 8-oxo-7,8-dihydro-2'-deoxyguanosine and Mutagenesis in Lung Epithelial Cells

**DOI:** 10.1155/omcl/8868348

**Published:** 2025-09-26

**Authors:** C. M. Lopez, D. C. Ramirez, S. E. Gomez Mejiba

**Affiliations:** ^1^Laboratory of Nutrition and Experimental Therapeutics, Multidisciplinary Institute for Biological Research-San Luis, CCT-San Luis, CONICET-National University of San Luis, San Luis 5700, San Luis, Argentina; ^2^Laboratory of Experimental and Translational Medicine, Multidisciplinary Institute for Biological Research-San Luis, CCT-San Luis, CONICET-National University of San Luis, San Luis 5700, San Luis, Argentina

**Keywords:** 8-oxo-dGuo, cell model, DNA-DMPO nitrone adduct, DNA radical, *hrpt* gene mutation, pulmonary neutrophilic inflammation

## Abstract

Pulmonary neutrophilic inflammation (PNI) is the recruitment and activation of neutrophils in the microvasculature with the release of myeloperoxidase (MPO) in the airways. Bystander epithelial cells can take up MPO, where it can generate HOCl. HOCl can react with DNA, generating DNA radicals, which then decay to produce several mutagenic end-oxidation products, such as 8-oxo-7,8-dihydro-2'-deoxyguanosine (8-oxo-dGuo). Herein, we aimed to test whether HOCl-induced DNA radicals precede DNA oxidation and mutagenesis in A549 human lung epithelial cells as an in vitro model that resembles PNI. Interestingly, by trapping HOCl-induced DNA radicals, the nitrone spin trap 5,5-dimethyl-1-pyrroline N-oxide (DMPO) blocks the formation of 8-oxo-dGuo and possibly other end-oxidation products, forming DNA-DMPO nitrone adducts. By preventing DNA oxidation, DMPO reduces the mutation of the hypoxanthine phosphoribosyl transferase (*hrpt*) gene, one of the genes most sensitive to oxidative damage. The transcription factor p53 is known as the master regulator of the cell response to genomic damage. By trapping DNA radicals, DMPO also blocks the translocation of p53 to the cell nucleus, suggesting that by trapping DNA radicals with DMPO, end-oxidation products are prevented, and the cell response to genomic damage is blunted. Trapping DNA radicals to reduce the accumulation of HOCl-induced mutagenic end-oxidation products will provide new therapeutic avenues to reduce genotoxic damage during PNI.

## 1. Introduction

Pulmonary neutrophilic inflammation (PNI) can be caused by biological, physical, mechanical, or metabolic irritants [1, 2]. Airway irritation results in the release of chemokines by tissues and resident macrophages that recruit neutrophils from the blood into the lung microvasculature [3]. Recruited neutrophils are activated with the production of superoxide radicals by NADPH oxidase-2 (NOX-2) [4] and the fusion of azurophil granules containing myeloperoxidase ([MPO], a donor hydrogen peroxide, oxidoreductase, and E.C. 1.11.1.7) with other components, including elastase, histones, and genomic DNA, to form neutrophil–extracellular traps (NETs) [5, 6]. In the extracellular milieu, extracellular DNAses releases NET-associated MPO, which can be taken up by surrounding tissue cells [6–8]. Inside cells, MPO can produce large amounts of HOCl [9, 10], a powerful and random oxidant that causes oxidative stress and exacerbates inflammation (discussed in [11]). At sites of inflammation, superoxide radicals, H_2_O_2_, and HOCl produced by MPO reduce the antioxidant capacity of cells and tissues, mainly through reactions with reduced glutathione (GSH), free protein sulfhydryl groups, and L-ascorbate [12]. This disbalance in favor of oxidants at sites of inflammation causes oxidative damage to macromolecules, a process known as oxidative stress [13]. Oxidative stress can in turn amplify the inflammatory process in airways exposed to irritants, leading to DNA damage, the inhibition of repair mechanisms, DNA mutations, and cell transformation [14].

Recruitment and activation of neutrophils in the airways is referred to as PNI [1], which is, from a biochemical standpoint, different from PNI, which is a term reserved for a histological pattern [2, 15]. Neutrophilic inflammation can result in oxidative stress and inflammation-related mutations, leading to cell transformation and carcinogenesis via oxidative modifications to genomic DNA and the oxidation of DNA damage-sensing and repair systems [16]. Previously, we reported that the addition of a bolus of HOCl or HOCl generated by the biochemical system MPO/H_2_O_2_/Cl^−^, can oxidize calf-thymus DNA, resulting in the formation of DNA radicals. Furthermore, in A549 cells loaded with MPO and in HL-60 cells that overexpress MPO, DNA radical formation can be determined by the use of the cell-permeable nitrone spin trap 5,5-dimethyl-1-pyrroline N-oxide (DMPO) and immuno-spin trapping (IST) as DNA-DMPO radical adducts [10]. A detailed protocol for measuring DNA radical formation using IST has been published [17] and reviewed previously [10]. Interestingly, DNA radical formation precedes 8-oxo-7,8-dihydro-2'-deoxyguanosine (8-oxo-dGuo) during hydroxyl radical and carbonate radical anion reactions with genomic DNA [18]. 8-oxo-dGuo is a molecular marker of DNA oxidation and potentially mutagenic transversions and transversions [19, 20].

Like GSH and L-ascorbate, DMPO is an antioxidant, but its mechanism of action is somewhat different. In fact, GSH and L-ascorbate are scavengers of free radicals. Upon reacting with radical sites in macromolecules or reactive oxygen species (ROS), scavengers are converted to free radicals [12]. DMPO is a nitrone spin trap [21]. Spin traps covalently bind to radical sites in radicalized macromolecules, forming radical adducts (which can be detected by electron spin resonance). This stops further macromolecule radical decay to macromolecule oxidation products (discussed in [10, 22]). In vivo, the reactions of DNA radicals with DMPO outcompete with high concentrations of antioxidants, oxygen, and the rate constants of these competing reactions (comprehensively discussed in [23]). During HOCl production in cells, the GSH and L-ascorbate concentrations decrease nitrone radical adducts [2, 24].

Neutrophilic inflammation is thought to cause genomic damage in tissues and may be involved in cell transformation [25–28]. Once MPO is taken up by surrounding epithelial cells [7], and at sites of inflammation where the concentration of H_2_O_2_—a cell permeable ROS—is in the range of 30–200 μM, the intracellular production of HOCl is possible [26]. Indeed, in HL-60 cells, MPO is present in the cell nucleus, whereas in A549 epithelial cells, it accumulates close to the nuclear envelope, and the intracellular concentrations of Cl^−^ are between 5 and 100 mM [9]. HOCl reacts with DNA to produce DNA chloramines that decay, forming DNA-(N)-centered radicals or DNA radicals [29, 30]. Unpaired electrons can be delocalized to other atoms in nucleotides, resulting in a wide range of oxidation and chlorination products (bases and sugars) [31]. In vitro, DNA radicals−and those formed when nucleotides, nucleosides, and bases react with HOCl have been previously observed using electron spin resonance [30]. The rate constants for the reactions of nucleotides and their components with HOCl have been reported [32].

DNA damage is sensed by the transcription factor p53, the master regulator of the cell response to DNA damage, which accumulates in the nucleus. Then, p53 binds to response elements in the regulatory region of genes encoding proteins involved in arresting the cell cycle until repair mechanisms are triggered [33–35]. On the other hand, the hypoxanthine phosphoribosyl transferase (*hrpt*) gene is one of the genes most sensitive to oxidative DNA damage [36]. The *hrpt* gene is mapped on the X chromosome of mammalian cells, and it is used as a model gene to investigate gene mutations in mammalian cell lines [37]. In addition, there is no doubt that neutrophilic inflammation may be involved in causing mutations and further cell transformation and carcinogenesis [16]. However, the mechanism connecting DNA radical formation and mutations remains to be fully elucidated.

Here, we show that HOCl produced by MPO inside A549 cells sequentially causes DNA radicalization, 8-oxo-dGuo formation, nuclear accumulation of p53, and mutation of the *hrpt* gene. DMPO blocks all these processes by trapping DNA radicals.

A preprint of this article was published in [38].

## 2. Methods

### 2.1. Treatment With Purified Human MPO

MPO was prepared as previously described [9]. Briefly, 100 μg of highly purified human MPO (Athens Research and Technology, cat# 16-14-130000) was dissolved in 200 μL of ultrapure water and dialyzed overnight against 2 L of 10 mM Chelex-sodium phosphate buffer, pH 7.4. To produce inactivated MPO, the enzyme was incubated with 100 μM KCN (KCN-MPO) or 500 μM 3-amino-1,2,4-triazole ([ATZ]-MPO) for 30 min at 37°C. Before the addition of MPO (native or inactivated) to the cell culture medium, the solution was sterilized by passing it through a 0.22 μm nylon syringe filter. UV-visible spectroscopy was used to determine the concentration, identity, and purity of the MPO. The Soret band with a peak at 430 nm (178,000 M^−1^ cm^−1^ for the MPO homodimer at pH 7.4) is characteristic of the specific heme-prosthetic group in MPO. The Rheinheitzahl value (*A*_430_/*A*_280_ ratio) from the UV-visible spectra provided an estimate of the purity of MPO relative to total protein. The preparations of native MPO used in our experiments had a Rheinheitzahl value of 0.8. MPO inactivation was measured by the oxidation of guaiacol to tetraguaiacol at 470 nm (ɛ_470 nm_ = 26.6 mM^−1^ cm^−1^) [9]. To adjust the MPO concentration, we performed protein measurements by using the BCA protein assay (QuantiPro BCA Assay Kit, cat#QPBCA, Sigma‒Aldrich, MI, USA), in which bovine serum albumin was used to prepare the standard curve.

### 2.2. The Biochemical Systems for the Generation of DNA Radicals

To determine the generation of DNA‒nitrone adducts in a biochemical system, we incubated calf thymus double-stranded deoxyribonucleic acid (dsDNA, ∼10,000–15,000 kDa), as its sodium salt (cat# D3664) with 5 μM MPO in 10 mM sodium phosphate buffer containing 140 mM Cl^−^, such as NaCl, pH 7.4. The generation of HOCl was started by the addition of a bolus of 50 μM H_2_O_2_ (ɛ_240_ = 43.6 M^−1^ cm^−1^). Fifteen minutes later, 50 μM DMPO (Dojindo Molecular Technologies Inc., Japan, Cat# D04810, ɛ_228_ = 7800 M^−1^ cm^−1^) was added to trap DNA radicals resulting from DNA–chloramine decay [30]. The total reaction volume was 100 μL. After 1 h of incubation, the reaction was stopped by the addition of 1 mM methionine. MPO inhibitors (KCN, 4-aminobenzoic acid hydrazide [ABAH, Cayman Chemicals, cat# 4845] or salicylhydroxamic acid [SHA, Sigma cat# S607]) or scavengers of HOCl (methionine or taurine [Tau]) were added 15 min before H_2_O_2_ addition.

### 2.3. A549 Cell Culture and Loading With MPO

The human lung type-2 epithelial cell line A549 (ATCC, Cat# CCL-185) was cultured in Ham's F12 medium (Lonza Biosciences, Cat# 12615F) containing 10% heat-inactivated fetal calf serum (FCS, Gibco, Ca#A3840101). For each experiment, A549 cells were cultured in 96-well or 6-well plates with 5 mm-round cover slides at the bottom. The cells were grown to 85% confluence before each experiment. A549 epithelial cells were loaded with MPO by incubation in fresh medium with 5% FCS and 10 nM native MPO or inactivated MPO (KCN-MPO or ATZ-MPO). After 24 h of incubation, the cells were rinsed with HBSS^−^ (HBSS without Ca^2+^ or Mg^2+^, Lonza, cat# 04--315Q) and incubated in HBSS^+^ with 50 μM H_2_O_2_ or HBBS^+^ containing 5 mM glucose (Glu), and HOCl generation started with 0.5 mIU/mL Glu oxidase (GO) for 15 min, followed by the addition or absence of 50 mM DMPO.

### 2.4. Neutrophil Isolation and Coculture With A549 Lung Epithelial Cells

The isolation and coculture of human neutrophils with A549 epithelial cells were performed as described previously [9]. Briefly, confluent monolayers of A549 cells and 10^6^ isolated human neutrophils in coculture were incubated in 2 mL of HBSS^+^ on round 5 mm cover slides in 6-well tissue culture plates at 37 °C. To generate NETs, we treated the cocultures with 100 ng/mL phorbol 12-myristate 13-acetate ([PMA]; Sigma, cat# P8139) for 1 h in an incubator. Finally, the monolayers were rinsed four times with HBSS^−^ before staining and confocal microscopy analyses.

### 2.5. Confocal Microscopy

For laser scanning confocal microscopy, the cells were cultured on 5 mm glass cover slides in 6-well plates. The immunostaining of MPO, p53, and histone H2B was performed following a procedure similar to that described in our previous study [9]. Briefly, after treatment, the slides were rinsed and fixed with 4% paraformaldehyde for 15 min at 37°C and then permeabilized with 0.2% Triton X-100 at room temperature for 5 min, followed by blocking with the Image-iT FX supersignal enhancer (Thermo Fisher Scientific, cat# I36933). The fixed cells were incubated overnight at 4°C with primary antibodies, washed, and then incubated with secondary antibodies at 37°C for 1 h. The primary antibodies used were anti-human p53 (Epitomics, cat# 1047-1, rabbit monoclonal anti-human p53, C-term, dilution 1:100), rabbit polyclonal anti-human MPO (Athens Research and Technology, cat# 01-14-130000, dilution, 1:500), and mouse monoclonal anti-human histone H2B (Abcam, clone 4G6, cat# H00008349-M06, dilution 1:100). The secondary antibodies used were goat anti-rabbit (Fc) conjugated to Alexa Fluor 488 or sheep anti-mouse IgG (Fc) conjugated to Texas Red. Finally, the slides were washed with PBS and mounted with Prolong Gold antifade mountant with 4,6-diamino-2-phenylindole (DAPI) to stain the nuclei (Thermo Fisher Scientific, cat# P36931), and the preparation was examined with a Leica SP2 MP confocal microscope with a 63 × 1.4 oil immersion objective. Single-plane or z-stack images were acquired and analyzed using the LSM 5 image examiner software.

### 2.6. Determination of H_2_O_2_-Triggered HOCl Production in MPO-Treated A549 Epithelial Cells

The amount of intracellular HOCl generated was determined using the luminol as a luminescent probe [39]. After the cells were loaded with MPO in a 96-well clear-bottom black microplate, the monolayers were rinsed, and 50 μL of 10 μM luminol (Sigma, cat# 123072) was added to 100 mM Chelex-sodium phosphate buffer, pH 7.4. When resveratrol ([Res], Sigma, cat# R5010) or Tau (Sigma, cat# T0625) was tested, they were added together with the luminol reagent. The intracellular generation of HOCl was started by adding 100 μL of 100 mM sodium phosphate buffer with 100 μM H_2_O_2_, and the luminescence was read in a microplate reader within 5 min of mixing the reagents at 15–22°C. When H_2_O_2_ was generated by the Glu/GO system, 1 mIU/mL GO (Sigma, cat# G7141) was added to the medium (HBSS^+^ with 5.6 mM Glu), and incubation was continued in the plate reader for 5 min at 37°C, followed by luminescence measurements. After the luminescence was read, the amount of dsDNA bound to the bottom of the plate was measured. Similar data were obtained with the use of the fluorescent probe 3'-p-aminophenyl) fluorescein (APF; Invitrogen, cat# A36003), which is a fairly specific probe for HOCl [40].

### 2.7. Quantification of Double-Stranded DNA Bound to Microtiter Plates

To quantify dsDNA bound to black microplates, we used DAPI, (Thermo Fisher Scientific, cat# D1306), which intercalates DNA and fluoresces (λ_ex_ = 345 nm/λ_em_ = 458 nm), as described previously [9].

### 2.8. Cell Viability and Glyceraldehyde-3-Phosphate Dehydrogenase (GAPDH) Activity Assays

Cell viability was determined using the trypan blue exclusion assay for adherent cells [41], 3-(4,5-dimethylthiazol-2-yl)-2,5-diphenyltetrazolium bromide tetrazolium (MTT) reduction assay [42], neutral red uptake assay [43], and lactic dehydrogenase (LDH) release assay [44]. One hundred percent and one percent viabilities were accomplished by incubating the cells under the same conditions as those used for the experimental groups with or without 1 mg/mL digitonin, respectively. GAPDH is a critical enzyme for cell survival and is extremely sensitive to HOCl-induced oxidative damage [11]. Thus, our experiments were complemented by the determination of GAPDH activity (mIU/mg protein) in homogenates to ensure early damage to energy metabolism. These assays were performed as described in our previous publication [11].

### 2.9. Cell Fractionation and Western Blot Analysis

To assess the nuclear accumulation of p53 in A549 epithelial cells loaded with or without MPO and treated with or without the H_2_O_2_-generating system (Glu/GO) in the presence or absence of DMPO, we performed cell fractionation and then western blot analysis. After 15 min of treatment, the cells were scraped, harvested, and fractionated as described by Bronfman et al. [45]. Briefly, after treatment, the cells were harvested, pelleted (20 s, 11,000 × *g*), and then treated with a buffer containing 0.25 sucrose, 3 mM imidazole, and 1 mg/mL digitonin, pH 7.4, at 4°C. The resuspended cells were then centrifuged for 20 seconds at 4°C. The supernatant contained the cytosolic proteins, whereas the pellet contained the nuclei. The protein concentrations in the cytosolic and nuclear fractions were quantified using the BCA assay. The protein concentration in the cytosolic and nuclear fractions was then adjusted to 10 mg/mL, and the proteins were separated by 10% SDS-PAGE. The separated proteins were blotted onto a nitrocellulose membrane using a buffer-less semidry apparatus (Bio-Rad). The presence of p53 in both fractions was detected using a rabbit monoclonal anti-human p53 antibody (Epitomics, cat#1047-1, 1:5000), with β-actin and histone H2B as loading controls for the cytosolic and nuclear fractions, respectively, which were used as loading controls. The polyclonal anti-β-actin antibody (Abcam, cat#ab8227) or the mouse monoclonal anti-human histone H2B antibody (Abcam, cat#H00008349--M06) were used at a dilution of 1:10,000 in washing buffer. Bound primary antibodies were detected using either a goat anti-rabbit IgG conjugated with horseradish peroxidase (HRP) (Abcam cat# ab6721) or a goat anti-mouse IgG-HRP (Abcam, cat# ab205719) secondary antibody. Both secondary antibodies were used at a dilution of 1:5000. After the membrane was rinsed, HRP-bound antibodies were detected using enhanced chemiluminescence and acquired with a C-DiGit^TM^ blot scanner (Li-COR Biotechnology). Band intensities were then measured with ImageJ software (https://imagej.net/ij/).

### 2.10. Extraction of DNA and Determination of 8-oxo-dGuo and DNA-DMPO Nitrone Adducts

After treatment, the cells were rinsed with HSSB^−^, and genomic DNA was isolated using the protocol we previously published [17]. The contents of 8-oxo-dGuo and DNA-DMPO nitrone adducts were determined by an enzyme-linked sorbent immunoassay (ELISA) as previously described [18].

### 2.11. 6-Thioguanine (6-TG) Mutagenesis Assay

The 6-TG mutagenesis assay can detect a wide range of chemicals capable of causing DNA damage that leads to gene mutation [37]. The 6-TG mutagenesis assay is based on the fact that mutations that destroy the functionality of the *hrpt* gene and/or protein are detected by positive selection using a toxic purine analog (6-TG). After 14 days of incubation with 10 μg/mL 6-TG (Sigma, cat# A4882), only the mutant cells survived and proliferated. A549 cells loaded with active MPO and treated with Glu or the Glu/GO system in the presence or absence of DMPO were screened for 6-TG resistance by using the trypan blue exclusion assay for adherent cells [41]. Supporting Information 1: Figure S4A provides a scheme of the workflow used for the 6-TG mutagenesis assay.

### 2.12. Statistics

The data are representative images or are shown as the means ± s.e.m. The Graph Pad Prism software package was used for statistical analysis. Differences between pairs were determined using the Student's *t* test, and differences between treatments and the control were determined via one-way analysis of variance with Dunnett's post hoc test. Differences were considered significant at *p* < 0.05.

## 3. Results

### 3.1. DNA Radical Formation by HOCl Produced by Active MPO

**Figure 1:**
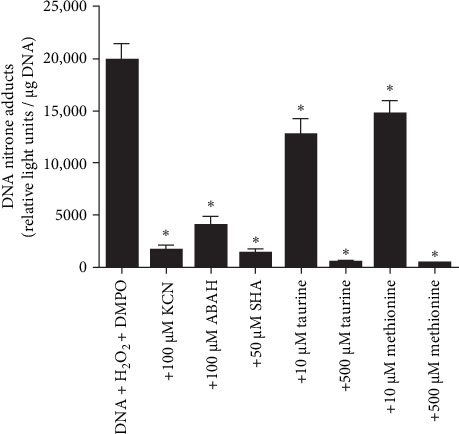
Calf-thymus DNA radicalization by the MPO/H_2_O_2_/Cl^−^ biochemical system and its measurement using immuno-spin trapping. ELISA for DNA-DMPO nitrone adducts formed when calf-thymus DNA was incubated with MPO in buffer containing physiological concentrations of Cl^−^, and HOCl formation started with H_2_O_2_. Potassium cyanide (KCN), 4-aminobenzoic acid hydrazide (ABAH), salicylhydroxamic acid (SHA), taurine, or methionine was added before starting HOCl formation with 50 μM H_2_O_2_. The data are shown as the mean values ± s.e.m. from three separate experiments performed in triplicate. Asterisks indicate *p*  < 0.05.

To show the HOCl-induced radicalization of DNA, we incubated calf thymus DNA with MPO and H_2_O_2_ for 15 min, followed by the addition of 50 mM DMPO to trap DNA radicals resulting from DNA-chloramine decay (Supporting Information 2: Figure S1). Once formed, DNA radicals can be scavenged by reduced GSH or L-ascorbate [9]. We measured DNA-DMPO nitrone adducts by ELISA (Figure 1). The addition of KCN or inhibitors of MPO, such as ABAH or SHA, resulted in a reduction in DNA-DMPO nitrone adduct formation. Tau and methionine reduce DNA radical formation by reacting with HOCl before it can react with DNA. Tau (4.8 × 10^5^ M^−1^ s^−1^) [46] and methionine (3.4 × 10^7^ M^−1^ s^−1^) [47] are two amino acids that, in excess, occur inside neutrophils, react quickly with HOCl at physiological pH, and thus protect DNA and other macromolecules from damage caused by this reactive halogen species.

### 3.2. MPO Produces HOCl and Causes DNA Radical Formation Inside Cells

**Figure 2:**
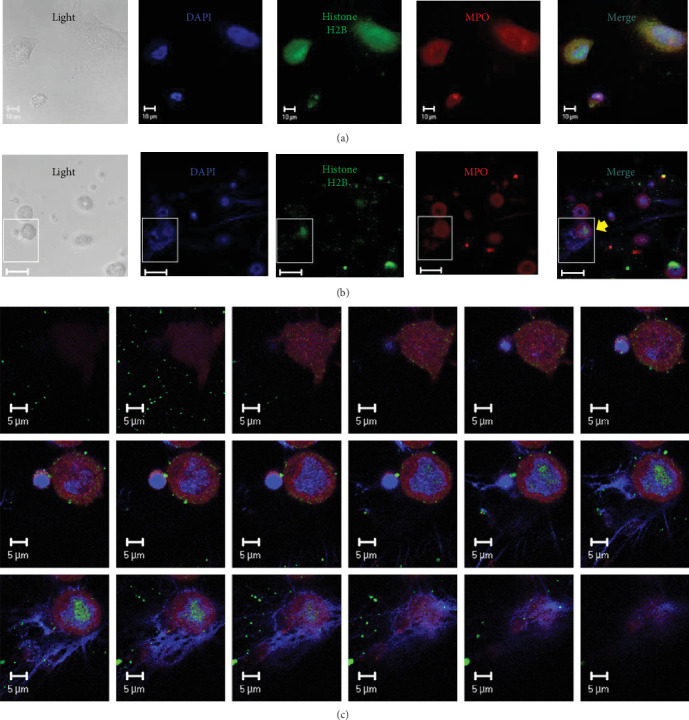
Coculture of A549 lung epithelial cells with PMA-activated neutrophils showing NETs in sequential planar images. (A) Scanning confocal microscopy images showing the localization of histone H2B (green), MPO (red), and genomic DNA (blue, DAPI) in A549 lung epithelial cells coincubated with neutrophils. The measurement bars represent 10 μM. (B) Same as A, but PMA was added to activated neutrophils to form NETs. The measurement bars represent 20 μM. (C) Eighteen z-stacks of planar images of the image marked with a yellow arrow in B. This image shows the interaction of a NET generated during neutrophil activation with one A549 cell. The measurement bars represent 5 μm. The images are representative of three individual experiments.

To simulate PNI, we incubated A549 lung epithelial cells without (Figure 2A) or with (Figure 2B) PMA-activated neutrophils. Neutrophil activation involves the formation of NETs in which MPO and other proteins, along with other nuclear components (histones and genomic DNA), are released, resulting in the formation of NET-like structures and the accumulation of MPO close to the nuclear membrane of A549 cells (Figure 2C). Z-stacks of images were used to observe NETs and the nuclear accumulation of MPO inside A549 cells.

**Figure 3:**
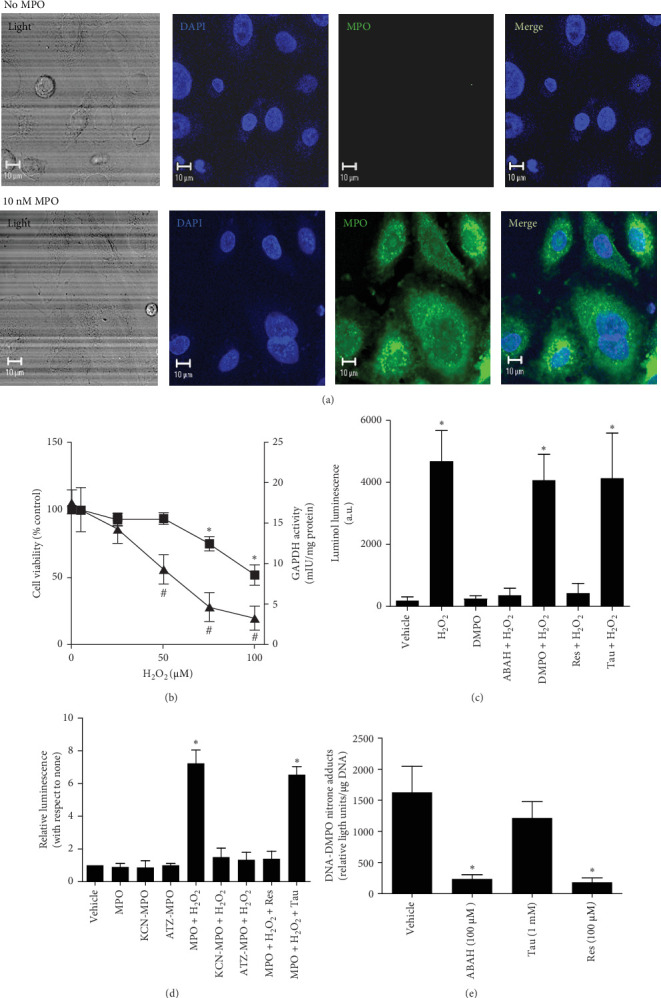
Intracellular production of HOCl by MPO inside A549 epithelial cells. (A) A representative laser confocal image showing the intracellular location of MPO inside A549 cells incubated without or with 10 nM purified human MPO. (B) Effects of intracellularly produced HOCl on cell viability (MTT reduction assay, closed squares) and GAPDH activity (closed triangles) in A549 cell homogenates. MPO-loaded A549 cells were incubated in HBSS^+^ containing 140 mM NaCl with different concentrations of H_2_O_2_ for 15 min. *⁣*^*∗*^ and # indicate *p*  < 0.05 with respect to the viability and GAPDH activity of the control (cells not treated with H_2_O_2_), respectively. (C) Luminol oxidation by intracellularly produced HOCl by active MPO. The cells were loaded with MPO and then incubated with luminol and DMPO, ABAH, Res or Tau. (D) Effect of the inactivation of MPO or scavenging of HOCl on luminol oxidation by intracellularly produced HOCl. Like C, but cells were preloaded with active (MPO) or preinactivated MPO (KCN-MPO and ATZ-MPO). (E) Effects of MPO inhibition or HOCl scavenging on DNA-DMPO adduct formation. MPO-preloaded A549 cells were incubated with DMPO, with or without an inhibitor of MPO (ABAH) or scavenger of HOCl (Tau or Res). DNA-DMPO nitrone adducts were measured by ELISA and referred to as DNA bound to the culture plate. In C–D, intracellular HOCl production was triggered by the addition of a bolus of 50 μM H_2_O_2_, and the vehicle was HBSS^+^ containing 140 mM NaCl. The data are presented as the mean values ± s.e.m. from four independent experiments. In panels C–E, an asterisk indicates *p*  < 0.05 with respect to cells not preloaded with MPO and incubated with vehicle (HBSS^+^ alone). a.u., arbitrary units.

To simulate PNI and the accumulation of MPO inside cells, we incubated A549 human lung epithelial cells with highly purified native human MPO and imaged its cell localization using confocal microscopy imaging (Figure 3A). This is a good model for studying HOCl-induced DNA radicalization in cells because any protein added to cell culture is taken up by cells [9]. We next measured the highest concentration of H_2_O_2_ that did not cause toxicity in A549 cells loaded with MPO (Figure 3B) after incubation for 15 min. The addition of a bolus of 50 μM H_2_O_2_ did not affect the viability of MPO-treated A549 cells, as assessed by the MTT assay. LDH release (Supporting Information 3: Figure S2A) and neutral red (Supporting Information 3: Figure S2B) assays yielded similar results. In any case, intracellularly generated HOCl can oxidize any protein depending on proximity, including GAPDH [11]—a highly ROS-sensitive enzyme critically involved in the glycolytic metabolism of Glu. A dose of 50 μM H_2_O_2_ caused a significant loss of enzyme activity (Figure 3B). A concentration of 100 μM H_2_O_2_ added to the culture of A549 epithelial cells without MPO did not affect either GAPDH activity or cell viability (Supporting Information 4: Figure S3), suggesting that the observed effects on cell viability and inactivation of GAPDH are caused by HOCl produced inside the cell.

To corroborate the production of HOCl inside the cells loaded with MPO and treated with H_2_O_2_, we used luminol, a cell-permeable fluorescent probe that reacts with reactive biochemical species [39]. To assess the effects of MPO inhibition or HOCl scavenging on HOCl-induced oxidation of luminol, A549 cells preloaded with MPO were incubated with or without vehicle or supplemented with or without 50 mM DMPO, 100 μM ABAH, 10 μM Res, or 1 mM Tau (Figure 3C). The intracellular production of HOCl inside cells was triggered by the addition of 50 μM H_2_O_2_ and was assessed with the use of luminol as a probe. To support these data, we tested whether Res and/or Tau can block the reaction with the probe. Tau is an amine that reacts with HOCl but does not cross cell membranes; however, Res is a trans-stilbene that crosses membranes and reacts quickly with HOCl before it reacts with luminol [9]. We observed that 1 mM Tau did not affect the luminescence signal, whereas 10 μM Res completely blocked the reaction of HOCl with the probe inside the cell (Figure 3C).

We then measured the production of HOCl inside A549 cells loaded with native and inactivated MPO after treatment with 50 μM H_2_O_2_. We observed that native and active MPO is needed to increase luminescence and that it was inhibited by Res but not by Tau (Figure 3D). Similarly, luminescence and nitrone adduct formation were inhibited when ABAH or Res, but not Tau, was added to the reaction medium (Figure 3E).

### 3.3. Trapping DNA Radicals With DMPO Prevents 8-oxo-dGuo Formation

**Figure 4:**
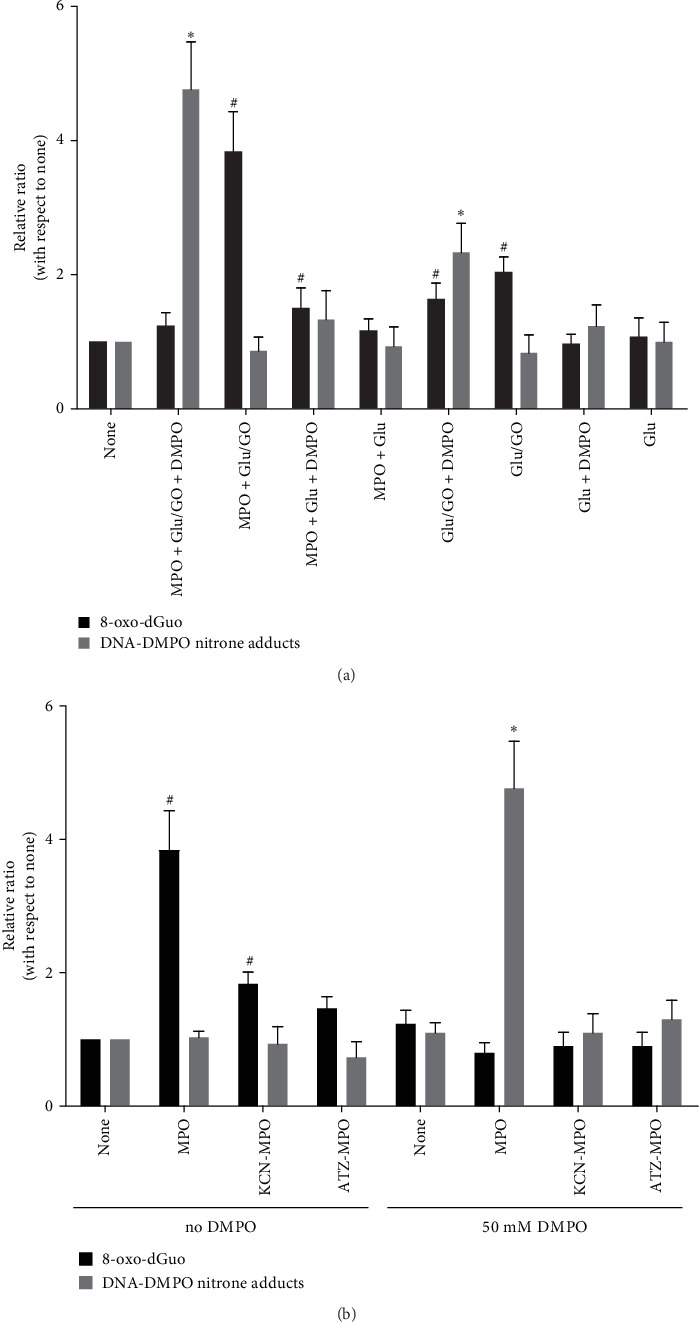
DMPO prevents the accumulation of 8-oxo-dGuo induced by intracellularly produced HOCl. (A) Quantification of 8-oxo-dGuo and DMPO-nitrone adducts in DNA isolated from A549 cells in which HOCl was intracellularly generated in the absence or presence of DMPO. The intracellular generation of HOCl was triggered by a continuous flow of H_2_O_2_ by the Glu/GO (5.6 mM Glu/1 mIU mL^−1^ GO) system. The reactions were stopped 30 min later by rinsing the cells with 100 μM resveratrol in Tris-buffered saline (pH 7.8), an amine buffer used to scavenge excess HOCl inside the cells. (B) Same as A, but the cells were loaded with native MPO or MPO that was previously inactivated (KCN-MPO or ATZ-MPO). Reactions were performed in either the presence or absence of 50 mM DMPO to measure DNA-DMPO nitrone adducts or 8-oxo-dGuo, respectively. The data are shown as the mean values ± s.e.m. from three independent experiments. The asterisk (*⁣*^*∗*^) indicates a difference (*p*  < 0.05) in DNA-DMPO nitrone adducts with respect to none (A549 cells not preloaded with MPO and incubated in HBSS^+^ alone). The pound symbol (#) indicates a difference in 8-oxo-dGuo with respect to none (i.e., A549 cells not preloaded with MPO and incubated with HBSS^+^ alone).

Previously, using a Fenton-like system (Cu^2+/^H_2_O_2_) to radicalize DNA, we reported that DMPO prevents 8-oxo-dGuo formation [18]. Similar experiments in HOCl-induced DNA radicals have not yet been reported. In this study, we used the Glu/GO system to produce a continuous flow of H_2_O_2_ to outcompete cell antioxidants (e.g., L-ascorbate and GSH) (Figure 4A). These experiments were performed in the presence or absence of DMPO to study the production of 8-oxo-dGuo and DNA-DMPO nitrone adducts, respectively. The production of H_2_O_2_ in cells loaded with MPO and in the absence of DMPO resulted in 8-oxo-dGuo production, whereas in the presence of DMPO, DNA radicals were trapped, and no 8-oxo-dGuo was produced (Figure 4A). To produce DNA-DMPO nitrone adducts in MPO-loaded A549 epithelial cells, H_2_O_2_ and DMPO were needed. We found that active MPO, Cl^−^, and H_2_O_2_ were needed to produce 8-oxo-dGuo and DNA-DMPO nitrone adducts in the absence and presence of DMPO, respectively (Figure 4B). Treating A549 cells preloaded with inactive MPO (KCN-MPO or ATZ-MPO) with the Glu/GO system did not result in 8-oxo-dG or DNA-DMPO nitrone adducts.

### 3.4. Trapping of DNA Radicals With DMPO Prevents *hrpt* Gene Mutation

**Figure 5:**
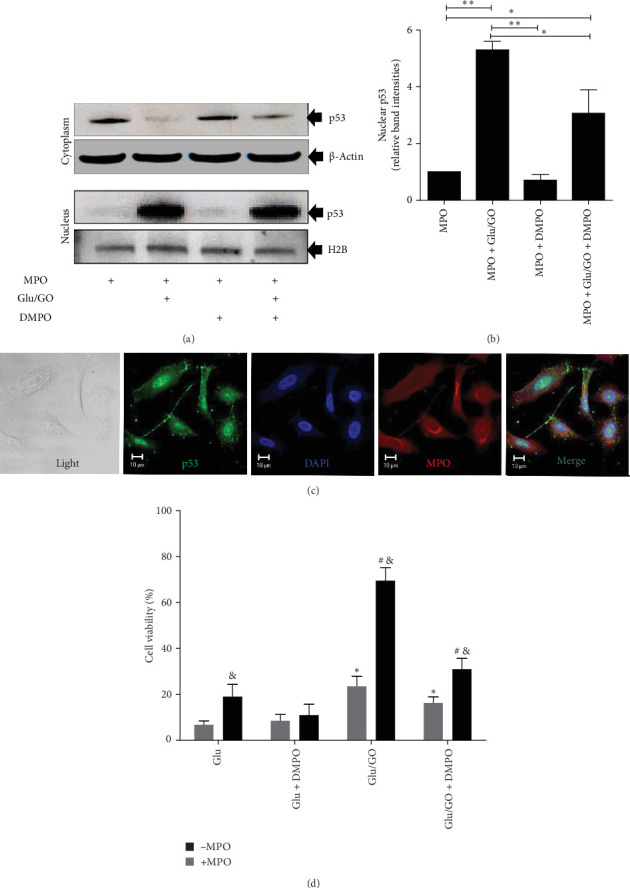
The intracellular generation of HOCl leads to the nuclear accumulation of p53. (A) Western blot analysis of p53 in the cytosolic and nuclear fractions of A549 epithelial cells preloaded with native MPO and then treated with the 5.6 mM Glu/1 mIU/mL GO system for 15 min in the presence or absence of DMPO. (B) Densitometric analysis of the p53 band intensities in the nuclear fraction shown in A using the via ImageJ software. Relative band intensities with respect to MPO preloaded cells (MPO) are depicted. The data are representative images or mean values ± s.e.m. from three independent experiments. *⁣*^*∗*^*p*  < 0.05 and *⁣*^*∗∗*^*p*  < 0.001 between the groups being compared, as indicated by the segments. (C) Planar confocal image showing changes in the compartmentalization of p53 in A549 lung epithelial cells treated as described in A. A representative image of three separate experiments is shown. (D) In-plate trypan blue exclusion assay for measurement of 6-TG-resistant cells (mutant cells) after 14 days of culture (Supporting Information 1: Figure S4A). The data are presented as the means ± s.e.m. from four independent experiments performed in triplicate. Symbols indicate *p*  < 0.05. *⁣*^*∗*^ compares the different groups (white bars) with respect to A549 cells without MPO and treated with glucose (Glu) alone; # compares MPO-loaded A549 groups (black bars) with respect to cells preloaded with MPO and treated with Glu; and ^&^ compares groups of cells preloaded with or without MPO (black vs. white bars) for each treatment.

Genomic damage is sensed by the genome guardian p53, a transcription factor that controls the expression of genes involved in cell cycle arrest [48]. In cells loaded with MPO and treated with H_2_O_2_, p53 accumulated in the nucleus (Figure 5a–c), suggesting that the genomic damage induced by intracellularly produced HOCl was sensed. Figure 5A shows a representative Western blot image showing the accumulation of p53 in the cytosolic fraction of A549 cells preloaded with native MPO and then exposed to the Glu/GO system. DMPO partially reduced p53 translocation inside the nucleus (Figure 5B). The confocal image shown in Figure 5C is consistent with the translocation of p53 inside the nucleus when HOCl is intracellularly produced by MPO.

The *hrpt* gene is one of the genes most sensitive to the oxidative damage caused by mutagens. Supporting Information 1: Figure S4A shows the workflow used to detect *hrpt* gene mutagenesis using the 6-TG reagent to select mutant cells. None of the cell treatments, loaded with or without MPO or with or without DMPO, caused significant cell death compared with cells treated with Glu only (Supporting Information 1: Figure S4B). After determination of cell viability, the medium in parallel plates was removed and replaced with fresh medium containing 10 μM 6-TG, after which the mixture was incubated for 14 days to select *hrpt* mutant cells. After this incubation, the cells were carefully harvested, and a trypan blue exclusion assay was performed to distinguish the death (6-TG-sensitive, nonmutant cells) of living cells (6-TG-resistant, mutant cells) (Figure 5D). These experiments revealed that treating MPO-preloaded A549 cells with a continuous flow of H_2_O_2_ resulted in the greatest generation of 6TG-resistant cells (Figure 5D). Mutagenesis of the *hrpt* gene, as well as the generation of 8-oxo-dGuo (Figure 4B), was prevented by DMPO when DNA radical formation occurred (Figure 5D).

## 4. Discussion

Oxidative stress caused by an inflammatory environment, such as airways exposed to air pollutants, can cause PNI-linked mutations, cell transformation, and lung cancer. Herein, we used an in vitro experimental model to study the role of DNA radical formation in mutagenesis during PNI. Our data are consistent with the critical role of DNA radical formation in HOCl-induced mutagenesis in lung epithelial cells. Indeed, if not trapped by DMPO, DNA radicals decay, among other DNA oxidation products, to 8-oxo-dGuo, causing mutations of the *hrpt* gene.

Upon pathogen infection or irritant exposure, local macrophages and other cells sense the insult and produce a wide panel of inflammatory mediators, such as cytokines and chemokines, that stimulate the nearby microvasculature and attract many neutrophils to migrate across the vascular wall and infiltrate into tissues [49, 50]. Tumor initiation, progression, and metastasis can be accelerated by oxidative damage to DNA during PNI. In addition, angioinvasion—a hallmark of aggressive tumor behavior—may be influenced by chronic oxidative injury, especially in epithelial tissues repeatedly exposed to inflammatory insults [51]. In fact, neutrophilic inflammation [1, 10] is an important acute response to irritation whose main purpose is to eliminate infection and enhance the adaptive immune response and tissue repair [50, 52]. However, when chronic or unresolved, inflammation can lead to chronic oxidative stress and damage pulmonary physiology, as observed in chronic obstructive pulmonary disease, which increases the risk of DNA damage and further cell transformation [53].

The role of HOCl produced by MPO in carcinogenesis has been proven in vitro and in experimental models [27]. Moreover, patients who express more MPO due to a single-nucleotide polymorphism in the promoter region (-463 G >A, rs#2333227) are the most susceptible to lung cancer [54], as are those with other chronic inflammatory diseases in which MPO plays a pathogenic role [55, 56]. The recent SARS-CoV-2 pandemic highlighted the importance of neutrophilic inflammation in genomic damage in airway cells [57]. Consequently, the study of the mechanism linking neutrophilic inflammation and oxidative DNA damage is of fundamental importance for reducing potential genotoxicity and mutagenicity in the lungs of COVID-19 patients.

**Figure 6:**
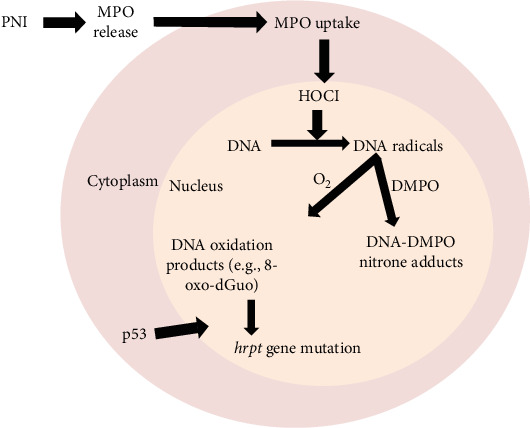
Scheme summarizing the main findings of this study. Pulmonary neutrophilic inflammation (PNI) in the lumen of airways causes the release of MPO. MPO is then taken up by airway epithelial cells. Inside airway epithelial cells, MPO produces HOCl, which upon reaction with genomic DNA causes DNA radical formation. DNA radicals quickly react with oxygen to give end-oxidation products, including 8-oxo-dGuo. 8-oxo-dGuo can lead to mutagenesis of the *hrpt* gene. The DNA damage-sensing mechanism is then activated by the translocation of p53 to the nucleus to trigger DNA repair mechanisms and determine cell fate. However, by trapping DNA radicals with DMPO, the formation of 8-oxodGuo, mutation of the *hrpt* gene, and p53 translocation into the nucleus are prevented.

MPO is the main enzyme contained in azurophilic granules of neutrophils that are released upon activation [58]. MPO can be taken up by cells in inflamed tissue, where HOCl production can damage the genome (Figure 6). MPO is the only animal peroxidase that, under physiological concentrations of halides and pH, produces HOCl [59, 60]. Depending on the H_2_O_2_ concentration, MPO operates via either a peroxidase cycle or a chlorinating cycle at high or low H_2_O_2_ concentrations, respectively [61]. The peroxidase cycle of MPO is important for radical production, whereas the chlorination cycle of MPO results in complex I oxidation of chloride anions into HOCl [62]. The chlorinating cycle of MPO is the most important cycle at sites of neutrophilic inflammation and results in increased chlorination of proteins, lipids, and nucleic acids [1, 62]. Physiological concentrations of Cl^−^ were used in our work to determine the production of HOCl in biochemical systems and A549 epithelial cells. Notably, the concentration of HOCl found at sites of neutrophilic inflammation has been estimated to be between 30 and 200 μM [63], which is higher than the concentrations we used in this study.

Neutrophil activation during PNI upon exposure to bacterial endotoxin involves the activation of NOX-2 and further NET formation [56, 64]. Damage caused by activated neutrophils in surrounding tissues involves oxidative modification and chlorination of proteins, lipids, and nucleic acids [55]. However, the neutrophil genome is protected against HOCl toxicity because of the high intracellular concentration of Tau (20–50 mM), which reacts faster with HOCl than any other biological target does [65].

The reaction of HOCl with DNA produces DNA chloramines that quickly decay, forming DNA radicals centered on nitrogen atoms [30]. Unpaired electrons can be transferred to other atoms in the structure of each nucleotide to damage the backbone, which results in DNA fragmentation, base chlorination (e.g., Cl-dG and Cl-dA), and other oxidation products, such as 8-oxo-dGuo [10, 16, 20, 31]. Res is a trans-stilbene that easily crosses cell membranes and reacts rapidly with HOCl before it reacts with luminol or genomic DNA [9]. Indeed, the rate constant for the one-electron oxidation of luminol by HOCl has been estimated to be approximately 10^−7^ s^−1^ (Personal communication, Dr. Michael Ashby, Department of Chemistry and Biochemistry, University of Oklahoma at Norman, OK, USA). For the first time, we found that by trapping DNA radicals with DMPO, HOCl-induced 8-oxo-dGuo formation in cells was blocked. We found similar results in trapping protein radicals with DMPO, observing that DMPO reduces HOCl-induced GAPDH fragmentation [11] and reduces carbonyls—one of the end products of free radical damage to proteins—in macrophages activated with lipopolysaccharide [66].

HOCl produced inside cells can not only deplete antioxidants, such as GSH and L-ascorbate, and damage DNA, but also oxidize critical signaling molecules involved in DNA damage-sensing and damage repair pathways. Upon oxidative DNA damage, p53 is translocated into the cell nucleus, where it binds to response elements located at regulatory sequences of genes involved in arresting the cell cycle until DNA repair or death is determined [33]. If repair mechanisms fail, mutant cells can escape this vigilance mechanism, and subsequently, cell transformation and carcinogenicity may occur [34]. We found that HOCl produced by active MPO inside A549 epithelial cells results in p53 translocation inside the nucleus. These results suggest that oxidative damage caused by intracellularly produced HOCl is sensed and that a proper response can be triggered.

Mutagenesis of the *hrpt* gene is one of the genes most sensitive to mutagenic insults [37]. To test whether HOCl triggers mutagenesis of the *hrpt* gene, a Glu/GO system was used to generate a nontoxic but continuous flow of H_2_O_2_ to overwhelm antioxidant defense mechanisms [9]. We found that intracellularly generated HOCl increased the number of 6-TG-resistant cells. These mutant cells, as well as the 8-oxo-dGuo content in their genomic DNA, were reduced if DNA radicals were trapped with DMPO [10]. Remarkably, our mutagenesis data are consistent with a nonmutagenic effect of DNA-DMPO nitrone adducts in the genome. We are still not sure whether, and if so, how, DNA-DMPO nitrone adducts can be repaired. However, Bhattacharjee et al. [67, 68] demonstrated using a model of macrophages treated with a hydroxyl radical-generating system that these DNA-DMPO nitrone adducts can be repaired over a short period, most likely through the base-excision repair pathway. They also reported that DNA repair enzymes modify DNA damage, including the removal of adducted DMPO, and that there are multiple and overlapping DNA repair pathways. This suggests that DMPO can block HOCl-induced mutagenesis, but we might be underestimating DNA-DMPO nitrone adduct formation [10].

Although our data clearly show a close link between HOCl, DNA radical formation, and mutation, it is important to consider some limitations regarding the use of DMPO, or its derivatives, as a potential therapeutic against HOCl-induced mutagenesis during PNI. Some of these limitations are as follows: (i) in this study, we used an in vitro model; however, validation of DNA radical formation and protection by DMPO has to be confirmed using primary epithelial cell cultures and in vivo models of pulmonary irritation. Similar studies using an in vivo model of acute respiratory distress syndrome and PNI-induced oxidative damage in mice exposed to endotoxin and whole bacteria are ongoing in our laboratories; (ii) while A549 cells offer a tractable model, data extrapolation to airway biology or lung cancer progression and aggressiveness requires caution; (iii) the fate of DNA-DMPO nitrone adducts and whether they can be sensed and repaired in still unknown; (iv) the potential off-target effects of DMPO or its metabolites in vivo could be carefully considered. Moreover, DMPO has never been used in humans. However, in rat studies, DMPO is excreted without metabolism in urine after 30 min of injection [69].

Taken together, our findings establish the causative role of transiently formed DNA radicals during mutagenesis produced by HOCl generated inside epithelial cells, which may explain PNI-induced carcinogenesis. Inhibitors of MPO, cell-permeable scavengers of HOCl (e.g., Res), or nitrone spin traps may help prevent the mutagenesis process caused by intracellularly produced HOCl (Figure 6). This information is useful for protecting the lung against oxidative stress- and inflammation-associated carcinogenesis caused by exposure of the airways to microbial and nonmicrobial air pollutants.

## Data Availability

The data supporting the findings of this study are available from the corresponding author upon reasonable request.

## Nomenclature

8-oxo-dGuo: 8-oxo-7,8-dihydro-2'-deoxyguanosine
6-TG: 6-thioguanine
ABAH: 4-aminobenzoic acid hydrazide
ATZ: 3-amino-1,2,4-triazole
DAPI: 4,6-diamidino-2-phenylindole
DMPO: 5,5-dimethyl-1-pyrroline-N oxide
Glu/GO: Glucose/glucose oxidase
HBSS^−^: Hank's buffered saline solution without Ca^2+^ and Mg^2+^
HBSS^+^: Hank's balanced saline solution with Ca^2+^ and Mg^2+^
IST: Immuno-spin trapping
hrpt: Hypoxanthine phosphorylbosyl transferase
MPO: Myeloperoxidase
NET: Neutrophil–extracellular trap
NOX-2: NADPH oxidase-2
PMA: Phorbol 12-myristate 13-acetate
PNI: Pulmonary neutrophilic inflammation
SHA: Salicylhydroxamic acid
res: Resveratrol
tau: Taurine.

## Supporting Information

Supporting Information 1 Figure S4: Workflow for the 6-TG selection assay in A549 epithelial cells in which HOCl is generated intracellularly. (A) Schematic workflow of the 6-TG selection assay in A549 cells preloaded with MPO. The intracellular production of HOCl was triggered by the Glu/GO system (Methods). (B) MTT reduction viability assay in cells that were treated as follows: loaded with 10 nM MPO, exposed to a flow of H_2_O_2_ generated by the 5.6 mM G/1 mIU/mL GO biochemical system with or without 50 mM DMPO for 15 min and then incubated for 7 days in fresh medium (workflow in panel A).

Supporting Information 2 Figure S1: Schematic pathway to produce DNA radicalization by HOCl generated by the MPO/H2O2/Cl- system. HOCl reacts with DNA to produce transient DNA chloramines that quickly decay, forming DNA-N-centered radicals. These radicals are trapped by DMPO-forming DNA‒DMPO radical adducts that decay, forming DMPO‒nitrone adducts ([9] for a recent review). These adducts are recognized by the anti-DMPO antibody. In cells, GSH and L-ascorbate can compete with DMPO for DNA radicals.

Supporting Information 3 Figure S2: Effects of intracellularly produced HOCl on cell viability. A549 lung epithelial cells were loaded (+MPO, closed squares) or not (−MPO, open squares) with MPO and incubated with different concentrations of H_2_O_2_ in HBSS^+^ containing 140 mM NaCl. After 30 min, the cells were rinsed to eliminate excess H_2_O_2_, and cell viability was assessed using the (A) a lactic dehydrogenase (LDH) release assay and (B) a neutral red uptake assay. Asterisks indicate *p* < 0.05 with respect to the absence of H_2_O_2_. The data are presented as the mean values +/− s.e.m. from four independent experiments.

Supporting Information 4 Figure S3: Effects of treatment of A549 epithelial cells with a bolus of H2O2 on cell viability and GAPDH activity. Effect of H2O2 on cell viability (MTT reduction assay, closed squares) and GAPDH activity (closed triangles) in A549 cell homogenates. A549 cells without MPO were incubated in HBSS^+^ containing 140 mM NaCl with different concentrations of H2O2 for 15 min. *⁣*^*∗*^ and # indicate *p* < 0.05 with respect to the viability and GAPDH activity of the control cells (with no H2O2 treatment), respectively. The data are presented as the mean values +/− s.e.m. from four independent experiments.
